# Constructing a Multidimensional Entrepreneurial Leadership Competency Model Amongst Nurse Leaders of New Hospitals in China: A Mixed-Methods Study

**DOI:** 10.1155/jonm/7626357

**Published:** 2025-10-06

**Authors:** Jing Gong, Binxu Yang, Lingxia Sun, Xintong Deng, Yingying Zhao, Fang He, Jing Zhou

**Affiliations:** ^1^Department of Nursing, The Second Affiliated Hospital of Zunyi Medical University, Zunyi, Guizhou, China; ^2^School of Nursing, Zunyi Medical University, Zunyi, Guizhou, China

## Abstract

**Objective:**

This study aims to assess and enhance the multidimensional entrepreneurial leadership competency model amongst nurse leaders of new hospitals in China.

**Background:**

As dynamically developing healthcare organisations, new hospitals face a more turbulent and uncertain environment compared with mature hospitals and often encounter uncertainties resulting from the complexity and ambiguity of their development process. The nurse leader's management philosophy, skill level and leadership style can greatly influence the construction of nursing teams, thus playing a key role in the management of new hospitals. Constructing a multidimensional nurse leader job competency model that meets the developmental qualities of new hospitals is thus critical to creating strategic value, improving nursing effectiveness and forming a competitive advantage in nursing.

**Method:**

This study adopted a mixed-research methodology and was conducted in two distinct phases. In the first phase, two rounds of in-depth semistructured interviews were performed with nurses, nurse managers, doctors and department heads in newly established hospitals. Participants were required to have worked in the new hospital for at least 1 year and to have been directly involved in clinical practice, nursing leadership or hospital management. A purposive sampling strategy was used to ensure diversity of roles and departments. The interviews were conducted by trained qualitative researchers; one acted as the facilitator, and the other took notes and managed audio recordings. After each round, thematic analysis was performed independently by two researchers, followed by discussion to reach consensus. The themes from both rounds were used to construct a competency model and develop a questionnaire. Grounded theory was applied to construct the dimensions and elements of the competency model. The model considered not only the elements of leadership for nurse leaders in new hospitals but also the developmental qualities of new hospitals. The second phase involved the development of a questionnaire based on the competency model identified in the first stage. Participants were recruited by convenience sampling. The respondents were nurses, nurse leaders, doctors and department heads of 2 new hospitals in Guizhou Province, with a total of 411 participants. Data collected from the questionnaires were used to test the reliability and validity of the model, the fit of the model and the consistency, accuracy and stability of its dimensions and elements.

**Results:**

The multidimensional nurse leader job competency model comprises 5 dimensions and 21 competency elements. After item analysis and exploratory factor analysis (EFA), 21 items were retained. The dimensions are establishing a shared vision (4 items, Cronbach's *α* was 0.850), problem-solving ability (5 items, Cronbach's *α* was 0.865), decision-making ability (4 items, Cronbach's *α* was 0.848), assuming uncertainty and risk (4 items, Cronbach's *α* was 0.850) and stimulating strategic innovation in nursing (4 items, Cronbach's *α* was 0.848). The total Cronbach's *α* was 0.939. Meanwhile, the cumulative variance explained by the five common factors extracted in accordance with customised choices in the EFA was 68.53%. In the validation factor analysis, *χ*^2^/*df*=1.235, RMSEA = 0.024, GFI = 0.952, CFI = 0.991, IFI = 0.991 and TLI = 0.989.

**Conclusions:**

The proposed multidimensional nurse leader job competency model shows good reliability, validity, fitness and scientificity and thus can be used for the assessment and training of nurse leaders in new hospitals. The five core dimensions were strongly supported by EFA and confirmatory factor analysis. These findings demonstrate that the model is theoretically sound and practically applicable for evaluating and developing nurse leadership competencies in clinical settings.

**Implications for Nursing Management:**

This study emphasises the importance of constructing a multidimensional job competency model for nurse leaders in new hospitals based on entrepreneurial leadership whilst considering the dynamic developmental qualities of new hospitals to facilitate the development of entrepreneurial leadership in the nursing field in China. The model shows strong applicability amongst nurse leaders in new hospitals, providing a structured approach to addressing diverse leadership challenges in evolving healthcare environments. Future assessment and training of nurse leaders should be guided by this model, with further research recommended to validate its effectiveness and refine its application in different nursing management contexts.

## 1. Background

New hospitals are organisations that provide high-quality and efficient healthcare services to the general public on a continuous basis, but there is still no exact standard regarding the time definition of newly built hospitals. Therefore, in this study, we try to define new hospitals on the basis of the meaning of start-ups as new healthcare organisations with legal representatives established within 42 months [1]. A mature hospital is a medical institution that has achieved a high level of synergistic development in multiple dimensions, such as healthcare, management, service, research and teaching and has a standardised management system and high-quality healthcare service capability [2]. In the context of China, new hospitals are typically characterised by rapid construction, policy-driven establishment (often in newly urbanised) and an urgent need to respond to regional medical service shortages. However, compared with mature hospitals, new hospitals have ‘nascent weaknesses' and uncertainties in the development process due to their lack of human capital and resources, such as insufficient senior clinical staff, limited interdisciplinary collaboration experience, underdeveloped medical information systems and incomplete emergency response mechanisms. Especially, the uncertainty of the general environment poses challenges and tests for new hospitals [3]. These issues create a highly complex and dynamic management environment, which increases the difficulty for leadership roles in such institutions. Although both new and mature hospitals are providing medical services for the health needs of the people, their development differs considerably: Mature hospitals are more interested in pursuing high-quality development on the basis of stability, whereas new hospitals are more concerned about how to quickly and efficiently establish facilities and service systems that meet the current demand for medical services [4].

New hospital nurse leaders play an important role in hospital management. In particular, nurse leaders in new hospitals serve as leaders, organisers, decision makers, participants and practitioners of clinical nursing and play a key role in the supervision and control of nursing quality in wards [5, 6]. Research shows that at the beginning of taking office, nurse leaders in new hospitals often face environmental adaptation, management thinking transformation and the integration of management knowledge and methods with the development characteristics of new hospitals, which are the difficulties and challenges for head nurses to be competent for the management position. At the same time, the dynamic changes in new hospitals introduce increasing requirements for the postcompetency of head nurses. Nurse leaders in new hospitals need to explore nursing opportunities, actively change and innovate, create nursing strategic value and improve nursing efficiency to build nursing competitive advantages [7, 8]. Some studies have demonstrated that the job competence of nurse leaders directly affects the entrepreneurial behaviours and quality of care of their subordinate nurses [9, 10]. However, a comprehensive framework for evaluating and cultivating the job competencies of nurse leaders in new hospitals remains lacking in existing literature. Most current competency models focus on general leadership, management or clinical skills, without adequately incorporating the adaptive, innovative and entrepreneurial qualities demanded in dynamic and uncertain new hospital environments. Determining how to select the best nurse leaders has therefore become a concern for nursing administrators in new hospitals. Unlike other leadership styles, entrepreneurial leadership can effectively respond to highly dynamic environments to help organisations build their competitive advantage.

Entrepreneurial leadership includes emerging leadership competencies such as creating vision, motivating subordinates, optimising corporate resource allocation, proactively seizing market opportunities and identifying and exploiting opportunities. A prestudy conducted from May 2022 to November 2022 involving 833 nurses from 3 new hospitals in Guizhou Province, China, revealed that high scores on the nurse leader's entrepreneurial leadership scale correspond to high scores of nursing teams in creativity, psychological safety, creative self-efficacy and knowledge sharing (*p* < 0.001) [11]. Nurse leaders with entrepreneurial leadership can enhance the trust of their subordinates through their own concepts, attitudes and behaviours; use different ways to stimulate their subordinates' creativity [12]; improve their nursing team members' self-confidence; evoke their high-level needs for the significance and value of their own work; allow them to express their views and ideas bravely. For instance, when faced with clinical nursing problems, nurse leaders with entrepreneurial leadership always come up with innovative solutions and share their knowledge and experience confidently. By demonstrating these qualities, these nurse leaders encourage their subordinates to strive in their work, improve their self-efficacy and creativity and create a work atmosphere that is conducive to innovation [13, 14].

This study aims to fill the research gap by constructing a multidimensional job competency model specifically tailored to the leadership roles in new hospitals. This model not only integrates classical management competencies but also incorporates innovative and entrepreneurial dimensions that are particularly relevant to the start-up nature of these institutions. The multidimensional job competency model for nurse leaders in new hospitals can guide their growth, assessment, training and scientific management. This model can improve the level of nursing management in new hospitals, enhance the core competitiveness of these institutions and promote their sustainable and high-quality development.

## 2. Methods

### 2.1. Research Design

This study adopted a two-phase mixed-methods design. Phase 1 used a qualitative approach to construct the dimensions and elements of a nurse leader competency model suitable for new hospitals. This phase involved two rounds of in-depth semistructured interviews with nurses, nurse leaders, doctors and department heads. The first round focused on initial model construction, and the second round tested for theoretical saturation. A questionnaire was then prepared based on the dimensions and elements of this model. Phase 2 employed a quantitative approach to develop a structured questionnaire based on the competency model derived from Phase 1 and validate it. The quantitative phase tested the model's reliability, validity, agreement, accuracy, stability and model fit.

### 2.2. Participants

#### 2.2.1. Qualitative-Phase Participants

Participants in this study were recruited through purposive sampling between March 2023 and September 2023 in newly established hospitals. Recruitment was carried out via internal hospital announcements and direct invitations by department heads, aiming to select individuals with relevant clinical and managerial experience. Purposive sampling was adopted to ensure a diverse range of perspectives from different professional roles and levels of responsibility. The selected participants included frontline nurses, nurse leaders (such as charge nurses and deputy head nurses), doctors and department heads. Semistructured interviews were conducted in two phases, following the principles of grounded theory. In the first phase, 29 participants were interviewed to explore and construct the preliminary dimensions and competency elements of the nurse leader competency model. In the second phase, six participants were interviewed to test the theoretical saturation of the developed model. The inclusion criteria were as follows: (1) the ability to communicate clearly and participate actively in interviews; (2) voluntary agreement to participate by signing an informed consent form; (3) at least 1 year of continuous working experience in a newly established hospital. For nurse leader participants, an inclusion criterion was added: (4) currently holding a nursing leadership position (e.g., charge nurse, deputy head nurse or above) with at least 1 year of formal leadership experience. This added criterion ensured that their responses were grounded in actual management responsibilities and practical experience. The exclusion criteria were as follows: (1) studying and training at a new hospital but not employed by the new hospital; (2) individuals who voluntarily withdrew or failed to complete the interviews.

#### 2.2.2. Quantitative-Phase Participants

For the quantitative phase, a cross-sectional survey was conducted from October 2023 to February 2024. Participants were recruited online using a convenience sampling method. A total of 411 individuals—including nurses, nurse leaders, doctors and department heads—from 2 new established hospitals in Guizhou Province were invited to participate. The inclusion criteria were as follows: (1) currently employed in a new hospital with at least 1 year of working experience; (2) voluntary agreement to participate with informed consent. The exclusion criteria were as follows: (1) studying and training at a new hospital but not employed by the new hospital; (2) participants who resigned during the study period (October 2023–February 2024); (3) respondents who provided random answers or completed less than two-thirds of the questionnaire items. The final sample size (*N* = 411) exceeded the commonly recommended minimum sample size requirements for both exploratory factor analysis (EFA) (at least 5 participants per item, *N* = 105) and confirmatory factor analysis (CFA) (at least 10 participants per item, *N* = 210), ensuring sufficient statistical power and robustness of the analysis. The sampling method was designed to allow broad representation from different roles within new hospitals to validate the competency model across professional boundaries.

### 2.3. Data Collection

#### 2.3.1. Qualitative Data Collection

Three days before the formal interviews, participants were contacted individually via phone or email to explain the purpose of the study, the confidentiality of the information collected and the logistics of the interviews. Participants received a copy of the interview outline and an informed consent form, which they returned either electronically or in hard copy prior to the interview. All interviews were conducted face to face in a private, quiet office or a designated meeting room within the hospital to ensure minimal disturbance and maintain confidentiality. Each interview was conducted by two researchers: one responsible for leading the interview and the other for taking notes and managing the recording equipment. With the participants' permission, all interviews were audio-recorded using a digital recorder to ensure accuracy of data collection. Several interview techniques, including paraphrasing, probing questions and clarification, were employed to enhance the depth and authenticity of participants' responses. Each interview lasted approximately 30–60 min. At the beginning of each session, participants completed a basic demographic form and signed an interview agreement. Throughout the research process, the confidentiality of the data was strictly maintained, and all audio files and transcripts were securely stored and were accessible only to the research team. The semistructured interview questions were reviewed and validated by three subject matter experts, namely, two senior nurse managers and one professor in nursing management, to ensure content relevance and clarity. An outline of the interviews is shown in Table 1.

#### 2.3.2. Quantitative Data Collection

In the quantitative phase, data were collected using a structured questionnaire based on a five-point Likert scale. The questionnaire consisted of two sections: The first section gathered demographic information (six items), namely, gender, job title, age, educational background, years of work experience and professional title. The second section assessed nurse leader competency elements in new hospitals, containing 5 dimensions with a total of 21 items. The scale consisted of 21 items rated on a five-point Likert scale (1 = very inconsistent, 2 = relatively inconsistent, 3 = generally consistent, 4 = relatively consistent and 5 = fully compliant) yielding a total score range of 21–105, with higher scores indicating higher levels of entrepreneurial leadership competency. A combination of paper-based and online surveys was employed. Paper questionnaires were distributed in person by the research team after obtaining written informed consent. Before administering the surveys, researchers explained the purpose and content of the study to each participant. For the online survey, participants were invited via the secure platform ‘Wen Juan Xing' (a widely used online survey tool in China). Participants completed the survey voluntarily via mobile phone or computer, and consent was implied through the completion and submission of the questionnaire. Each participant was invited to complete the survey only once, either online or on paper, to avoid any duplication of data. Confidentiality and anonymity were emphasised throughout the process, and participants' personal data were securely stored.

### 2.4. Data Analysis

#### 2.4.1. Qualitative Data Analysis

Two independent coders, namely, the first author and a qualitative expert, coded and analysed the interview texts using NVivo. The analysis followed Zagan's theory of open coding, axial (spindle) coding and selective coding, involving multiple readings, coding, summarising and refinement of source materials. In the first round of interviews, themes were initially generated through open coding, which helped identify core concepts and subcategories related to nurse leader competencies. These preliminary categories were further refined during axial coding, in which relationships between themes were established to develop competency dimensions. In the second round of interviews, these themes were re-evaluated to test for theoretical saturation. The researchers reviewed whether any new themes emerged and whether the existing dimensions and elements adequately captured the participants' perspectives. No significant new codes emerged in round two, confirming that saturation was reached. Based on the gathered data, the relevant dimensions and elements of the proposed competency model were constructed.

The questionnaire used in the quantitative phase was directly derived from the final competency model developed through this qualitative analysis. Specifically, the themes and subthemes extracted from coding were translated into questionnaire items under each competency dimension, ensuring that each item reflected the core competencies identified from participants' interviews. During the semistructured interviews, two researchers were present. One researcher, typically the first author, was responsible for leading the interview, asking questions and encouraging open discussion. The second researcher managed note-taking and operated the audiorecording equipment. Both researchers worked together during the analysis to ensure consistency in interpreting participant responses.

Two experts, one with a PhD in nursing and the other with a PhD in English, were invited to translate the interview transcripts independently. The researcher compared and integrated the two translations and presented the first draft to the two experts after discussion. Two academics fluent in Chinese and English—one a nursing expert—were then asked to review the recordings, check the transcripts line by line and back-translate the text from English to Chinese. The researcher discussed the back-translated results with the participants and made adjustments in collaboration with the back-translators wherever necessary to ensure consistency with the participants' intended meanings. All data were collected anonymously. Instead of using participants' real names, each was assigned a code denoting their position. For instance, NM denotes nurse leader, N denotes nurse, D denotes doctor and DD denotes department head.

#### 2.4.2. Quantitative Data Analysis

SPSS version 29.0 was used to test the reliability and validity of the proposed competency model, validating the agreement, accuracy and stability of its dimensions and competency elements under different conditions. Descriptive statistics, reliability analysis (Cronbach's alpha) and EFA were first conducted to test internal consistency and factor structure. Amos 27.0 was then used for CFA to assess the goodness of fit between the hypothesised five-dimensional competency model and the actual survey data. Model fit indicators such as *χ*^2^/df, RMSEA, CFI and TLI were used to evaluate structural validity.

### 2.5. Ethical Considerations

The study protocols were reviewed by the Second Affiliated Hospital of Zunyi Medical University (Ethics No. KYLL-2023-019). During the interviews and questionnaire survey, the participants were informed about the purpose and significance of the study, and the researcher emphasised the anonymity of their responses. After obtaining their consent to participate, these participants were asked to sign an informed consent form. The research process followed the principles of informed consent, voluntariness, fairness, confidentiality and helpfulness. Additionally, we confirm that all participating hospitals provided written agreement for participation, including consent for their anonymised data to be used in publications. The names of the hospitals have been kept confidential as per institutional policy and ethical approval conditions.

## 3. Results

### 3.1. Qualitative Results

#### 3.1.1. Demographic Profile

A total of 35 participants were interviewed, with the interview recordings lasting for nearly 1200 min. More than 110,000 words of primary data were collected, of which 90,000 words were acquired during the first round and 20,000 words during the second round.  Table 2 presents the basic information of the interview participants. These participants were aged between 27 and 53 years and were working across several departments, including medicine, surgery, oncology, obstetrics and gynaecology, ICU and emergency medicine. All these participants held a bachelor's degree or higher. Although the primary aim of this study focused on nurse leaders, the participant group for the two rounds of interviews included a diverse range of healthcare professionals—such as nursing managers, doctors, department heads and subordinate nurses—to gather broad perspectives on competency needs. This heterogeneity in participant roles may have introduced variability but enriched the data from different management levels.

#### 3.1.2. Open Coding

A total of 29 interview texts were analysed sentence by sentence on the basis of grounded theory, and key words or phrases were tagged to form the initial categories to be used in the subsequent coding process for building a specific coding structure. This coding process and its results provided a solid foundation for this study.  Table 3 presents a selection of raw utterances that were used to demonstrate the coding categorisation adopted in this study.

#### 3.1.3. Axial and Selective Coding

The relationships amongst the initial categories were analysed, and the repeated concepts were clustered into main categories. These main categories were further refined to extract the dimensions and elements of the proposed competency model.  Table 4 presents the overall coding results.

#### 3.1.4. Theoretical Saturation Test and Model Construction

**Figure 1:**
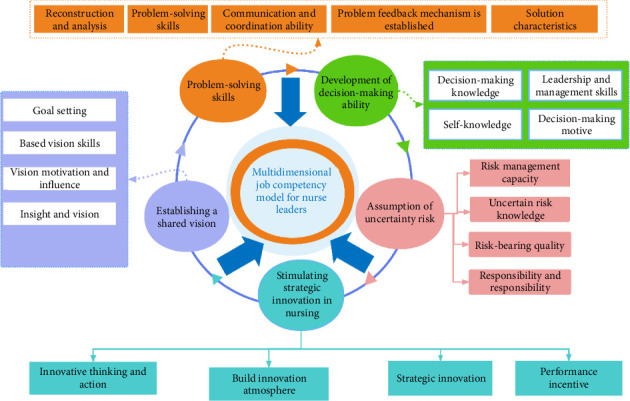
Multidimensional competency model based on entrepreneurial leadership for nurse leaders in new hospitals. Note: Entrepreneurial leadership underpins all five dimensions of the model, including innovation, decision-making and future-oriented abilities, and serves as the theoretical framework guiding the development of competencies.

When three-level rooted coding is performed using empirical data collected from other participants and no new concepts, categories and relationships emerge, the theory is said to have reached saturation [15]. The textual data collected from the six participants in the second round of interviews were used as the basis for the theoretical saturation test in this study. These six participants served different work positions in their new hospitals. The previous three-level coding steps were performed on their data, and no new concepts and categories emerged. Therefore, the preliminary theoretical saturation of the constructed model was reached based on the available qualitative data (Figure 1). However, the final validation of the theoretical constructs will be further supported through CFA in the quantitative phase.

### 3.2. Quantitative Research Results

#### 3.2.1. Description of Participants

A total of 411 nurses, nurse leaders, doctors and department directors participated in the questionnaire survey. In this sample, females slightly outnumbered males (67.64%). These participants were aged between 20 and 65 years (with 45.50% aged between 31 and 50 years) and held either bachelor's or master's degrees. Those participants working for 1–5 years, 6–10 years, 11–20 years and more than 21 years accounted for 25.55%, 31.63%, 29.68% and 13.14% of the sample, respectively. Their competence levels were mainly proficient and expert, as shown in Table 5.

#### 3.2.2. Reliability and Validity Analyses

A reliability analysis was conducted on the data collected from the 411 survey participants to evaluate the internal consistency of the proposed competency model. Cronbach's *α* coefficients were calculated for each dimension and for the overall scale. Before the final reliability scores were computed, item–total correlations and Cronbach's *α* if items were deleted were examined. Items with a corrected item–total correlation lower than 0.3 or that significantly reduced internal consistency were considered for removal. However, after analysis, all items were retained as they met the reliability criteria. The final model consisted of 21 items across 5 dimensions, each demonstrating high internal consistency. The reliability coefficients, along with mean scores and standard deviations for each dimension, are presented in Table 6. The reliability for the overall scale, calculated across all 21 items, was 0.939, indicating excellent internal consistency and strong psychometric properties. These results confirmed that the proposed competency model is a reliable tool for assessing nurse leaders' entrepreneurial leadership competencies in newly established hospitals.

#### 3.2.3. Validity Analysis

##### 3.2.3.1. Exploratory Factor Analysis

The Kaiser–Meyer–Olkin value was 0.95, whilst Bartlett's sphericity test was significant (*χ*^2^ = 4269.159; *p* < 0.001), suggesting that the sample data are suitable for an EFA. For the EFA, principal component analysis with varimax rotation was applied. The number of factors was determined based on eigenvalues > 1, scree plot inspection and theoretical interpretability [16]. CFA was subsequently conducted using Amos 27.0, following established guidelines. Therefore, principal component analysis was employed to conduct factor analysis on 21 observed variables (competency elements), yielding five principal components. Five common factors with initial eigenvalues exceeding 1 were extracted, accounting for 68.529% of the cumulative variance and individually explained 15.686%, 13.402%, 13.179%, 13.144% and 13.118% of the variance. After the six iterations converged, five common factors were extracted based on the proposed competency model. Generally, a variance contribution exceeding 50% is considered sufficient to establish acceptable factor validity. The loadings of these factors were greater than 0.6, implying that the factor extraction results agreed well with theoretical expectations and can effectively explain the variability in the data. Therefore, the proposed competency model has excellent explanatory power (Table 7).

##### 3.2.3.2. Confirmatory Factor Analysis

###### 3.2.3.2.1. Convergent Validity

CFA was performed on the five dimensions, whose CR values were above 0.7 and mean variance extraction volume AVEs were all above 0.5. Hence, these dimensions have convergent validity (Table 8).

###### 3.2.3.2.2. Distinguishing Validity

Table 9 shows that the correlation coefficients amongst the variables were below the AVE square root values of each latent variable, indicating a good discriminant validity between each latent variable.

#### 3.2.4. Model Fit

The model fit was tested using Amos 27.0. The obtained indices (chi-square = 221.010, DF = 179 and CMIN/DF = 1.235) indicated a good model fit (Table 10).

## 4. Discussion

The core driving force behind improving the quality, efficiency and motivation of medical and health personnel comes from the hospital staff themselves. Talent management represents a breakthrough point in management [17]. The job competency model, as an efficient tool for analysing and evaluating human resources, serves as the core foundation of the human resource management system. Nurse leaders play a crucial role in hospital management. The quality of their work directly affects the medical experience of patients and the nursing ecology of their hospitals [18]. These nurse leaders need to identify the direction of the dynamic development of new hospitals, recognise fleeting development opportunities, stimulate the work effectiveness of their nursing team members, promote innovation, guide their subordinates in overcoming difficulties and encourage them to work hard towards realising the development vision of their new hospitals [19].

Entrepreneurial leadership shows great potential in developing nursing teams in new hospitals. Therefore, this study builds a competency model for the position of nurse leaders in new hospitals based on entrepreneurial leadership. This model encompasses five dimensions, namely, establishing a shared vision, problem-solving skills development of decision-making capacity, assumption of uncertainty risk, stimulating strategic innovation in nursing. By highlighting the key competencies required for nurse leaders working in newly established hospitals, this model can help new hospitals select the right candidate for such position. This candidate should be able to lead nursing teams in carrying out high-quality nursing work, improving patient satisfaction and enhancing the hospital's reputation.

Establishing a shared vision means that the individual goals of nurses and the goals of the nursing team are combined to form a common goal that is generally accepted and agreed upon by the members of the nursing team [20]. Leaders and team members agree that having a shared vision provides a clear direction and a strong sense of purpose in their practice. This vision also helps them stay on track, set their priorities and work towards the same goals. In the early stage of hospital development, the lack of a shared vision amongst medical staff may lead to poor work efficiency, limited teamwork, low service quality, high staff turnover and challenges in hospital culture construction [21]. As team leaders, organisers and policy makers, nurse leaders in new hospitals are responsible for leading, guiding and coordinating nursing teams. The establishment of a shared vision enhances the cohesion amongst nursing teams, which acts as the driving force behind the sustainable development of hospitals [22].

Establishing a shared vision of dimensions covers the motivation and goal setting, as well as the establishment of vision, vision effect, insight and vision competence elements. Nurses in new hospitals, owing to their own dynamics, can easily feel anxiety and uncertainty, which may reduce their motivation and enthusiasm. Goal setting by nurse leaders can help clarify the work direction and goals of subordinate nurses and help them address their work challenges, thus building their motivation and enthusiasm and improving their work ability [23]. Therefore, nurse leaders in new hospitals should fully understand the development trend of their respective departments and identify the present advantages to establish a shared vision in their teams [24]. New hospitals need to establish their own brand image in their development process and transmit their values to the outside world. Nurse leaders are thus required to consider the positive societal impacts of having a shared vision [25]. They may build insights into the advantages of each nurse in their teams to further understand their respective advantages and disadvantages and to provide personalised guidance [26].

As public health institutions, new hospitals have greatly improved the equity and accessibility of medical and health care. However, these hospitals face great uncertainty in their development, generally have younger staff than other mature hospitals, have weak scientific research and innovation and lack a sound forming system. Nurse leaders in new hospitals should therefore be able to effectively and quickly solve problems to ensure the smooth operations and steady development of their institutions [27, 28]. Problem-solving ability includes five competency elements, namely, problem reconstruction and analysis, problem-solving skills, communication and coordination, establishment of problem feedback mechanism and problem-solving characteristics, all of which aim to ensure that nurse leaders in new hospitals have excellent ability to cope with complex situations. Firstly, problem reconstruction and analysis require nurse leaders to identify the priorities in their management work, analyse and evaluate problems in depth and achieve resource integration, which are helpful in quickly identifying key problems in medical services and understanding the nature of these problems [29]. Secondly, problem-solving skills require nurse leaders to have a wide range of business knowledge, to be able to use information technology and to efficiently respond to problems [30, 31]. Thirdly, communication and coordination elements require nurse leaders to prevent and solve conflicts within and outside their teams; establish smooth communication and coordination amongst patients, doctors and nurses; promote collaborations between doctors and nurses across all levels in the hospital and provide efficient and comprehensive medical services for patients [32]. Fourthly, by establishing a problem feedback mechanism, nurse leaders can understand the problems encountered by subordinate nurses in their work and provide them with support, which can help improve their work efficiency and satisfaction [33]. Finally, nurse leaders should be able to maintain a positive attitude when dealing with problems. A study on emotional contagion theory reveals that the emotional stability of team leaders can affect creativity within their teams [34].

Nurse leaders are also responsible for the daily operations and management of care teams, and their decision-making ability can contribute to the efficient delivery of medical services [35]. The decision-making ability dimension in the proposed competency model covers four elements, namely, decision-making knowledge, leading the management team, self-awareness and decision-making motivation [36]. Firstly, decision-making knowledge requires nurse leaders in new hospitals to possess the relevant knowledge when facing different problems or groups. They need to understand the characteristics, needs and diseases of different patient groups to effectively guide nurses, adjust nursing plans and improve their quality of care. They should be able to make informed decisions on the basis of the available information, respond quickly in case of emergencies and ensure the continuity and quality of medical services [37]. Secondly, leadership management teams require nurse leaders to make fair decisions, balance the interests of all parties, encourage cooperation, promote team development and improve the accuracy and execution of decisions [37]. Thirdly, nurse leaders need to have a clear value orientation, a well-defined core value system and a strong and decisive decision-making style to encourage their teams to work towards the goal of their hospitals [38]. Finally, developing decision motivations emphasises financial returns and competitive advantage. Given that the new medical industry requires huge financial investments, economic returns are amongst the key drivers of the construction and operation of new hospitals [39]. Emphasising financial returns in decision making can help nurse leaders in new hospitals optimise their limited resources and improve the economic benefits of nursing.

The development of new hospitals faces several challenges, including the changing needs of patients, lack of funding and limited training of medical personnel, all of which may affect the construction and operation of these hospitals [40]. Solving the uncertainties surrounding these hospitals requires collaboration with management teams. Nurse leaders who bear uncertain risks can enhance the psychological safety of nurses and build their innovation ability and creative self-efficacy [41]. In the proposed competency model, uncertainty risk includes the four elements of risk management, risk knowledge, risk-bearing quality and responsibility bearing, which are designed to ensure that nurse leaders can effectively deal with uncertainties. Firstly, risk management requires nurse leaders to mobilise and guide their teams, ensure their ability to work as a whole and keep their emotions under control when dealing with uncertainties. Nurse leaders should also be able to make decisions quickly, coordinate the team resources effectively and maintain the normal operations of hospitals in the face of internal or external risks [42]. Secondly, risk knowledge includes understanding laws and regulations and having a professional knowledge of risk assessment and control [43]. Nurse leaders should be able to understand the laws and regulations in their field to maintain the normal operations of their hospitals. They should be able to accurately evaluate and control potential risks to reduce uncertainties [44]. Thirdly, risk-bearing literacy requires nurse leaders to adapt to situation changes, take responsibility and establish self-norms. They should be flexible, willing to take responsibility and establish and follow specifications to ensure the safe, stable and sustainable development of hospitals. Fourthly, nurse leaders should take the responsibilities of managing wards and training technical leaders and young nurses. The leadership behaviour of these leaders can directly affect the quality of their departments [45].

Innovation in nursing strategies refers to the development and implementation of nursing strategies by nurse leaders. These leaders are expected to break away from traditional frameworks and models by introducing new ways of thinking, methods or tools and by forming a nursing strategy system with unique competitive advantages. This innovation focuses not only on the current development of nursing teams but also on their future growth and sustainable development. Stimulating nursing strategy innovation covers four elements, namely, innovative thinking and action, creating an innovative atmosphere, strategic characteristics and performance incentive, through which nurse leaders can promote innovation in hospital care, improve the performance of nursing teams and ensure the quality and efficiency of medical services [46]. Firstly, innovative thinking and action require nurse leaders to make financial decisions, demonstrate an excellent management ability, encourage continuous education and knowledge sharing amongst teams and ensure the psychological safety of teams, all of which contribute to the creation of a positive team atmosphere [47]. Secondly, to create an innovative atmosphere, nurse leaders should encourage and support the use of innovative technology through performance evaluations and incentives. They should also create an open and supportive environment to facilitate the implementation of innovative ideas [48]. Thirdly, nurse leaders must bear hardships and stand hard work. They need to demonstrate patience, tolerance and effective time management skills to improve work efficiency [49]. Finally, performance incentive is a management strategy that encourages the innovative behaviour of employees through the provision of rewards and incentives. In the context of this study, reward plans can encourage nurses to propose innovative ideas, solve problems and demonstrate improved work efficiency and quality.

Comparative analysis was performed between our proposed multidimensional entrepreneurial leadership competency model and existing entrepreneurial leadership frameworks, such as those proposed by Gupta et al. [50], Renko et al. [49] and Bagheri and Pihie [51]. These classic models largely emphasise general entrepreneurial leadership traits such as innovativeness, proactiveness, risk-taking and opportunity recognition, predominantly in business or corporate contexts. However, such models are typically abstract and context neutral and often lack operational guidance for high-stakes, team-based environments like healthcare.

Compared with existing entrepreneurial leadership models, our model is specifically contextualised for nurse leaders in new hospitals [52]. Whilst it retains core entrepreneurial elements, it further integrates competencies critical to clinical leadership, such as shared vision, evidence-based decision-making and nursing-specific innovation. This tailoring to the healthcare environment addresses the unique challenges of uncertainty, team-based coordination and patient-centred care. The proposed model not only aligns with but also extends existing entrepreneurial leadership theories by translating abstract entrepreneurial traits into a structured, clinically relevant competency framework. It contributes theoretically and practically to the understanding and development of entrepreneurial leadership in nursing.

The above findings confirm the scientific nature and reliability of multidimensional nurse leader job competence. The proposed competency model does not require a perfect nurse leader but is committed to helping new nurse leaders grow along with their new hospitals, flexibly respond to various challenges, eliminate the dominant management logic in their minds, differentiate their work from routine nursing management, maximise the innovative vitality of subordinate nurses and guide their teams towards shared goals.

## 5. Implications for Nursing Management

This study emphasises the importance of constructing a multidimensional job competency model for nurse leaders in new hospitals based on entrepreneurial leadership whilst considering the dynamic developmental qualities of new hospitals to facilitate the development of entrepreneurial leadership in the nursing field in China. The model demonstrates strong applicability amongst nurse leaders by addressing the key competencies required in rapidly evolving clinical settings, particularly in newly established hospitals. It can serve as a practical framework for guiding recruitment, evaluation and targeted leadership development programmes. This model should be incorporated in future assessment and training of nurse leaders in new hospitals to cope with leadership issues and challenges in the workplace. Further research is recommended to validate the model in diverse hospital settings and explore its long-term effect on leadership performance and organisational outcomes in nursing management.

## 6. Limitations

This study constructed a competency model for nurse leaders in new hospitals based on entrepreneurial leadership and examined its fit via validated factor analyses. Although the results confirmed the good fit of this model, the data were all collected in China. Future studies should examine the applicability of this model to nursing management practices in new hospitals in other countries to further prove its validity and reliability and promote its use in practice. Although this study focused on constructing a competency model for nurse leaders in new hospitals, the quantitative survey phase also included staff nurses, doctors and department directors. This approach aimed to obtain a multidimensional evaluation of leadership competencies from diverse professional perspectives. However, the inclusion of participants with different roles and responsibilities may have introduced response variability, which should be considered when interpreting the findings.

## 7. Conclusions

Understanding the competencies required from nurse leaders can help new hospitals formulate strategies for cultivating competent nurse leaders. This study incorporated 21 competencies and divided them into 5 dimensions. The proposed competency model focuses on building a shared vision, quickly solving problems in the development of new hospitals, making the right decisions, taking calculated risks and inspiring innovation amongst nursing staff. This model considers the development characteristics of new hospitals, stresses the coordinated matching of ‘people' with ‘positions' and avoids phenomena such as seniority, skills and performance assessments, thus providing a valuable reference for the selection and assessment of nurse leaders in new hospitals. The model showed strong internal consistency (Cronbach's *α*=0.848–0.865, total *α*=0.939) and excellent construct validity (*χ*^2^/df=1.235, RMSEA = 0.024, CFI = 0.991), confirming its scientific reliability. Therefore, this competency model can serve as a practical and evidence-based tool for leadership development, performance evaluation and talent planning in newly established hospitals.

## Figures and Tables

**Table 1:** Outline of the interview protocol for the position of nurse leaders in new hospitals.

Outline of the interview
1. What qualities do you think nurse leaders should have, and why?
2. Describe in a few words, as accurately and comprehensively as possible, the leader of the nursing team in your department.
3. Before answering the next question, let me briefly explain the difference between new and mature hospitals. New hospitals are recently established healthcare institutions (within 42 months), often still developing their systems and teams; by contrast, mature hospitals are well established and stable in structure, processes and human resources. Now, do you think that the competency of nurse leaders in new hospitals differs from that of nurse leaders in mature hospitals? Please explain your answer in detail.
4. Which qualities or abilities of nurse leaders do you think can lead to the success of nursing teams in new hospitals?
5. Entrepreneurial leadership refers to a leadership style that emphasises innovation, proactiveness, opportunity recognition and adaptability to change. On the basis of this understanding, what competency elements do you think should be included in the competency model of nurse leaders with entrepreneurial leadership?
6. In actual nursing management work, what kind of management methods do you want the nurse leaders in your department to adopt?
7. In your opinion, what qualities or abilities should a nurse leader in a new hospital possess to be considered successful, given the hospital's fast-paced and uncertain environment?
8. If you are an administrator in a new hospital, what would be your criteria when selecting nurse leaders?

**Table 2:** Characteristics of the participants in the two rounds of interviews (*N* = 35).

Characteristics	Profile	Number (%)
Position	Nursing managers	10 (28.6%)
	Nurses	10 (28.6%)
	Doctor	8 (22.8%)
	Department head	7 (20%)

Gender	Male	13 (37.3%)
	Female	22 (62.7%)

Age (years)	20–30	8 (22.9%)
	31–40	17 (48.6%)
	41–50	9 (25.7%)
	51–60	1 (2.8%)

Clinical experience (years)	1–5	6 (17.1%)
	6–10	12 (34.5%)
	11–15	6 (17.1%)
	16–20	8 (22.8%)
	≥ 21	3 (8.5%)

Interview time (min)	30–40	10 (28.6%)
	41–50	13 (37.1%)
	51–60	12 (34.3%)

**Table 3:** Open coding category examples.

Primary source statements	Initial category
Guide the nurses clear their goals, clear team goals.	Clear goals
As a head nurse, are you sure you want to clear a trend in the development of the whole department.	Understanding of the trend of development in the department
No matter what encounter the first affirmation is to guarantee the safety of the patients, and then is to minimize the risk of the patient.	Identify priorities
The head nurse's information technology level and the teaching level is better than other general head nurse.	Information technology skills
I think the head nurse in the professional field is a new hospital to achieve a high level, to participate in the management of a department.	Professional skills and knowledge
I think the doctor and nurse self should be a family, so the head nurse or a division director must be harmonious to deal with, is the medical relationship must be harmonious rapport.	Medical communication and coordination
For example, to other hospital nursing management to learn some good management experience, this is what we need to have.	Seek professional advice
Original new head nurse in the hospital, he may also enter the new head nurse is the process of transformation, she certainly doesn't see anything, so I feel or want to listen to all aspects of the voice.	Decision-making information collection
Nurse scheduling requirements, for example, in the face of nurse's emotional things, she can more quickly find ways to solve the problem.	Emotional awareness and management
A good leader, I think whether it is a nurse or a director, should have responsibility and when the bear	Responsibility
The nurse will worry, because they make a mistake, and then can be abandoned by the whole team, or be eliminated. So, giving them the psychological security is very important.	Maintenance team psychological safety
Besides subject development itself, along with the development of the era, some features can be linked to our medical, I think we need to absorb the era have entrusted to one of your advantages.	Open adoption and authorisation
The head nurse's ability to plan as a whole and his patience careful, these are very important, in addition to professional quality, is the head nurse's tolerance.	Patience and tolerance

**Table 4:** Main and selection coding results.

Core category	Main category	Initial category
A1 Establishing a shared vision	A11 Goal setting	Clear goals
		Identify the core values
	A12 Based vision skills	Understanding of the trend of development in the department
		Identify the advantages and shortcoming
		Implementation of the plan
	A13 Vision motivation and influence	Positive social impact
		Vision flexibility
	A14 Insight and vision	Looking ahead
		Superior foresight

A2 Problem-solving skills	A21 Reconstruction and analysis	Identify priorities
		Problem analysis and evaluation
		Resource conformity assessment
	A22 Problem-solving skills	Clinical competence
		Information technology competence
		Education and scientific research strength
		Professional skills and knowledge
	A23 Communication and coordination ability	Conflict prevention and solution
		Patient communication and coordination
		Handling interpersonal relationships
		Medical communication and coordination
	A24 Problem feedback mechanism	Learn and improve
		Seek professional advice
	A25 Solution characteristics	Emotional poise and stability
		Details of the control
		Positive upward
		Be flexible
		Awareness of self-improvement

A3 Development of decision-making ability	A31 Decision-making knowledge	Controls the decision-making results
		Decision-making information collection
		Emergency treatment
	A32 Leadership and management skills	Fair and just
		Ability to lead
		Weigh the pros and cons
		Group decision
		Solidarity and collaboration
		Experience and seniority
	A33 Self-knowledge	Values orientation
		Decisions driven
		Quotient of emotion
		Executive force
	A34 Decision-making motive	Economic return
		Competitive advantage

A4 Assumption of uncertainty risk	A41 Risk management capacity	Mobilisation and guidance
		Work as a whole and reshaping
		Emotional awareness and management
	A42 Uncertain risk knowledge	Laws and regulations
		Risk assessment and control
	A43 Risk-bearing quality	Adapting to scenery changes
		Self-regulation
	A44 Responsibility and responsibility	Dare to take responsibilities
		Dare to face responsibilities

A5 Stimulating strategic innovation in nursing	A51 Innovative thinking and action	Financial decision-making management
		Encourage continuous education
		Encourage knowledge sharing
		Maintenance team psychological safety
	A52 Build an innovation atmosphere	Encourage and support innovative technology
		Open adoption and authorisation
	A53 Strategic innovation	Bear hardships and stand hard work
		Patience and tolerance
		Good affinity
		Time management
	A54 Performance incentive	Performance evaluation
		Build a system of rewards and punishments

**Table 5:** Characteristics of the survey participants (*N* = 411).

Characteristics	Profile	Frequency (*n*)	Percentage (%)
Gender	Male	133	32.36
	Female	278	67.64

Position	Nurse	136	33.09
	Head nurse	129	31.39
	Doctor	82	19.95
	Department head	64	15.57

Age (years)	20–30	102	24.82
	31–40	128	31.14
	41–50	85	20.68
	≥ 51	96	23.36

Final education	Bachelor's degree and below	5	1.22
	Bachelor's degree	214	52.06
	Master's degree	151	36.74
	Doctoral degree or above	41	9.98

Working experience	1–5 years	105	25.55
	6–10 years	130	31.63
	11–20 years	122	29.68
	21 years	54	13.14

Competence level	Novice	110	26.76
	Proficient	142	34.55
	Expert	159	38.69

*Note:* Participants with a bachelor's degree and below were primarily frontline clinical staff, such as staff nurses, who were included to provide insights into the leadership competencies of nurse leaders they interact with. They were not employed in managerial or leadership positions.

**Table 6:** Reliability analysis of the entrepreneurial leadership competency model for nurse leaders.

Construct	Items	Cronbach's *α*	M	SD
Establishing a shared vision	4	0.850	3.89	1.61
Problem-solving skills	5	0.865	3.93	1.11
Development of decision-making capacity	4	0.848	3.92	1.11
Assumption of uncertainty risk	4	0.850	3.94	1.17
Stimulating strategic innovation in nursing	4	0.848	3.96	1.15

**Table 7:** Rotated factor loading coefficients of the proposed competency model.

Item	A1	A2	A3	A4	A5
A11 Goal setting	**0.730**	0.233	0.157	0.184	0.192
A12 Establishing vision skills	**0.770**	0.172	0.220	0.194	0.127
A13 Vision motivation and influence	**0.697**	0.174	0.177	0.225	0.266
A14 Insight and vision	**0.701**	0.203	0.176	0.179	0.315
A21 Reconstruction and analysis	0.053	**0.758**	0.186	0.200	0.187
A22 Problem-solving thinking	0.181	**0.704**	0.248	0.213	0.139
A23 Communication and coordination	0.159	**0.701**	0.105	0.249	0.222
A24 Establish a problem feedback mechanism	0.189	**0.712**	0.116	0.156	0.198
A25 Solution characteristics	0.333	**0.721**	0.180	0.175	0.120
A31 Decision-making knowledge	0.221	0.169	**0.721**	0.184	0.265
A32 Leadership management team	0.110	0.099	**0.812**	0.130	0.162
A33 Self-perception	0.206	0.230	**0.690**	0.192	0.233
A34 Decision-making motive	0.205	0.276	**0.723**	0.210	0.134
A41 Risk management ability	0.159	0.265	0.137	**0.726**	0.241
A42 Uncertain risk knowledge	0.243	0.232	0.182	**0.713**	0.173
A43 Risk-taking literacy	0.161	0.210	0.172	**0.747**	0.185
A44 Responsibility and tolerance	0.225	0.216	0.236	**0.720**	0.158
A51 Innovative thinking and action	0.279	0.221	0.185	0.264	**0.674**
A52 Creating a climate of innovation	0.221	0.214	0.165	0.167	**0.722**
A53 Strategic innovation	0.179	0.173	0.217	0.181	**0.743**
A54 Performance incentives	0.196	0.210	0.239	0.173	**0.728**

*Note:* Establishing a shared vision, A1; Problem-solving skills, A2; Development of decision-making capacity, A3; Assumption of uncertainty risk, A4; Stimulating strategic innovation in nursing, A5. A1 includes A11–A14, A2 includes A21–A25, A3 includes A31–A34, A4 includes A41–A44 and A5 includes A51–A54. The loadings of these factors were greater than 0.6, implying that the factor extraction results agreed well with theoretical expectations and can effectively explain the variability in the data. Therefore, the proposed competency model has excellent explanatory power.

**Table 8:** Regression parameters and elements of the proposed competency model.

Item	Dimension		Standardized path coefficient	S.E.	C.R.	*p*	CR	AVE
A11 Goal setting	<--	A1	0.746				0.850	0.586
A12 Establishing vision skills	<--	A1	0.759	0.066	14.815	^∗∗∗^		
A13 Vision motivation and influence	<--	A1	0.766	0.066	14.960	^∗∗∗^		
A14 Insight and vision	<--	A1	0.791	0.067	15.439	^∗∗∗^		
A21 Reconstruction and analysis	<--	A2	0.745				0.865	0.562
A22 Problem-solving thinking	<--	A2	0.759	0.068	14.951	^∗∗∗^		
A23 Communication and coordination	<--	A2	0.740	0.069	14.567	^∗∗∗^		
A24 Establish a problem feedback mechanism	<--	A2	0.713	0.067	14.008	^∗∗∗^		
A25 Solution characteristics	<--	A2	0.788	0.071	15.535	^∗∗∗^		
A31 Decision-making knowledge	<--	A3	0.792				0.848	0.584
A32 Leadership management team	<--	A3	0.710	0.057	14.524	^∗∗∗^		
A33 Self-perception	<--	A3	0.768	0.063	15.883	^∗∗∗^		
A34 Decision-making motive	<--	A3	0.783	0.063	16.214	^∗∗∗^		
A41 Risk management ability	<--	A4	0.771				0.851	0.587
A42 Uncertain risk knowledge	<--	A4	0.772	0.063	15.600	^∗∗∗^		
A43 Risk-taking literacy	<--	A4	0.746	0.062	15.035	^∗∗∗^		
A44 Responsibility and tolerance	<--	A4	0.776	0.063	15.694	^∗∗∗^		
A51 Innovative thinking and action	<--	A5	0.791				0.848	0.583
A52 Creating a climate of innovation	<--	A5	0.741	0.058	15.371	^∗∗∗^		
A53 Strategic innovation	<--	A5	0.746	0.055	15.471	^∗∗∗^		
A54 Performance incentives	<--	A5	0.774	0.057	16.142	^∗∗∗^		

*Note:* Establishing a shared vision, A1; Problem-solving skills, A2; Development of decision-making capacity, A3; Assumption of uncertainty risk, A4; Stimulating strategic innovation in nursing, A5.

^∗∗∗^ *p* < 0.001.

**Table 9:** Correlation of the dimensions of the proposed competency model.

	A1	A2	A3	A4	A5
A1 Establishing a shared vision	**0.766**				
A2 Problem-solving skills	0.669	**0.750**			
A3 Development of decision-making capacity	0.665	0.646	**0.764**		
A4 Assumption of uncertainty risk	0.691	0.713	0.659	**0.766**	
A5 Stimulating strategic innovation in nursing	0.745	0.670	0.699	0.696	**0.764**

*Note:* The correlation coefficients among all variables in this study were lower than the square root of the AVE for each latent variable. Therefore, it can be concluded that the discriminant validity among the latent variables is good.

**Table 10:** Fitness of the proposed competency model.

Model	CMIN/DF	RMSEA	CFI	GFI	IFI	TLI
Measured value	1.235	0.024	0.991	0.938	0.991	0.989
Standard value	< 5.00	< 0.08	> 0.90	> 0.90	> 0.90	> 0.90

*Note:* CMIN/DF: chi-square value/degrees of freedom.

Abbreviations: CFI, comparative fit index; GFI, goodness-of-fit index; IFI, incremental fit index; RMSEA, root-mean-square error of approximation; TLI, Tucker–Lewis's index.

## Data Availability

The data that support the findings of this study are available upon request from the corresponding author. These data are not publicly available because of privacy or ethical considerations.
